# Identification of potential immune-related hub genes in Parkinson’s disease based on machine learning and development and validation of a diagnostic classification model

**DOI:** 10.1371/journal.pone.0294984

**Published:** 2023-12-05

**Authors:** Guanghao Xin, Jingyan Niu, Qinghua Tian, Yanchi Fu, Lixia Chen, Tingting Yi, Kuo Tian, Xuesong Sun, Na Wang, Jianjian Wang, Huixue Zhang, Lihua Wang

**Affiliations:** 1 Department of Neurology, The Second Affiliated Hospital of Harbin Medical University, City Harbin, Province Heilongjiang, China; 2 Department of Neurology, The 962 Hospital of the Chinese People’s Liberation Army Joint Logistic Support Force, City Harbin, Province Heilongjiang, China; Fondazione Don Carlo Gnocchi, ITALY

## Abstract

**Background:**

Parkinson’s disease is the second most common neurodegenerative disease in the world. However, current diagnostic methods are still limited, and available treatments can only mitigate the symptoms of the disease, not reverse it at the root. The immune function has been identified as playing a role in PD, but the exact mechanism is unknown. This study aimed to search for potential immune-related hub genes in Parkinson’s disease, find relevant immune infiltration patterns, and develop a categorical diagnostic model.

**Methods:**

We downloaded the GSE8397 dataset from the GEO database, which contains gene expression microarray data for 15 healthy human SN samples and 24 PD patient SN samples. Screening for PD-related DEGs using WGCNA and differential expression analysis. These PD-related DEGs were analyzed for GO and KEGG enrichment. Subsequently, hub genes (*dld*, *dlk*1, *iars* and *ttd*19) were screened by LASSO and mSVM-RFE machine learning algorithms. We used the ssGSEA algorithm to calculate and evaluate the differences in nigrostriatal immune cell types in the GSE8397 dataset. The association between *dld*, *dlk*1, *iars* and *ttc*19 and 28 immune cells was investigated. Using the GSEA and GSVA algorithms, we analyzed the biological functions associated with immune-related hub genes. Establishment of a ceRNA regulatory network for immune-related hub genes. Finally, a logistic regression model was used to develop a PD classification diagnostic model, and the accuracy of the model was verified in three independent data sets. The three independent datasets are GES49036 (containing 8 healthy human nigrostriatal tissue samples and 15 PD patient nigrostriatal tissue samples), GSE20292 (containing 18 healthy human nigrostriatal tissue samples and 11 PD patient nigrostriatal tissue samples) and GSE7621 (containing 9 healthy human nigrostriatal tissue samples and 16 PD patient nigrostriatal tissue samples).

**Results:**

Ultimately, we screened for four immune-related Parkinson’s disease hub genes. Among them, the AUC values of *dlk*1, *dld* and *ttc*19 in GSE8397 and three other independent external datasets were all greater than 0.7, indicating that these three genes have a certain level of accuracy. The *iars* gene had an AUC value greater than 0.7 in GES8397 and one independent external data while the AUC values in the other two independent external data sets ranged between 0.5 and 0.7. These results suggest that *iars* also has some research value. We successfully constructed a categorical diagnostic model based on these four immune-related Parkinson’s disease hub genes, and the AUC values of the joint diagnostic model were greater than 0.9 in both GSE8397 and three independent external datasets. These results indicate that the categorical diagnostic model has a good ability to distinguish between healthy individuals and Parkinson’s disease patients. In addition, ceRNA networks reveal complex regulatory relationships based on immune-related hub genes.

**Conclusion:**

In this study, four immune-related PD hub genes (*dld*, *dlk*1, *iars* and *ttd*19) were obtained. A reliable diagnostic model for PD classification was developed. This study provides algorithmic-level support to explore the immune-related mechanisms of PD and the prediction of immune-related drug targets.

## 1. Introduction

Parkinson’s disease(PD) is the second most common neurodegenerative disease globally, commonly found in middle-aged and older adults, slightly more men than women [1, 2]. More than 6 million people worldwide had PD in 2016, and the incidence of Parkinson’s disease is gradually increasing. Parkinson’s disease is expected to reach 10 million people by 2030 [3].

Currently, a definitive diagnosis of Parkinson’s disease needs to be made in a specialized hospital or by a specialized clinician [4]. This is difficult for most people with Parkinson’s disease who live in underdeveloped or less developed areas. Some studies have shown that the corresponding pathological processes and neurodegeneration begin 10–20 years before the main motor symptoms [5–7]. The latest prodromal diagnostic criteria for Parkinson’s disease were released in 2019 and are thought to hold promise for early identification of disease progression in the preclinical phase of PD [8]. However, the implementation of these tests requires specialized personnel and appropriate testing equipment, so they are not widely used in clinical practice. Therefore, simplifying the detection process, early identification, and diagnosis of Parkinson’s disease has become an urgent issue before us.

Pathophysiology suggests that Parkinson’s disease may result from a complex interplay of abnormal alpha-synuclein aggregation, dysfunction of mitochondrial, lysosomal or vesicular transport, synaptic transport problems, and neuroinflammation [9]. Together, these disease mechanisms lead to the accelerated death of major dopaminergic neurons [4]. The immune function also plays a complex role in Parkinson’s disease [10]. On the other hand, the immune function also exhibits neuroprotective effects [10]. In addition, alterations in the intestinal environment can also affect inflammation and the accumulation of α-synuclein [11]. Clarifying the interaction of PD in central and peripheral immunity will help us to identify patients with PD prodromal stage early and carry out early interventions. Arlehamn et al. found that immune activity against α-synuclein in monocytes of peripheral blood was associated with preclinical PD [12]. This suggests that monitoring this biomarker in a high-risk population has the potential for early detection of Parkinson’s disease. The most important pathological feature of Parkinson’s disease is the loss of dopaminergic neurons in the substantia nigra. Immune activity in the substantia nigra (SN) may be the "final common pathway" of immunity throughout the disease [13–15].

**Fig 1:**
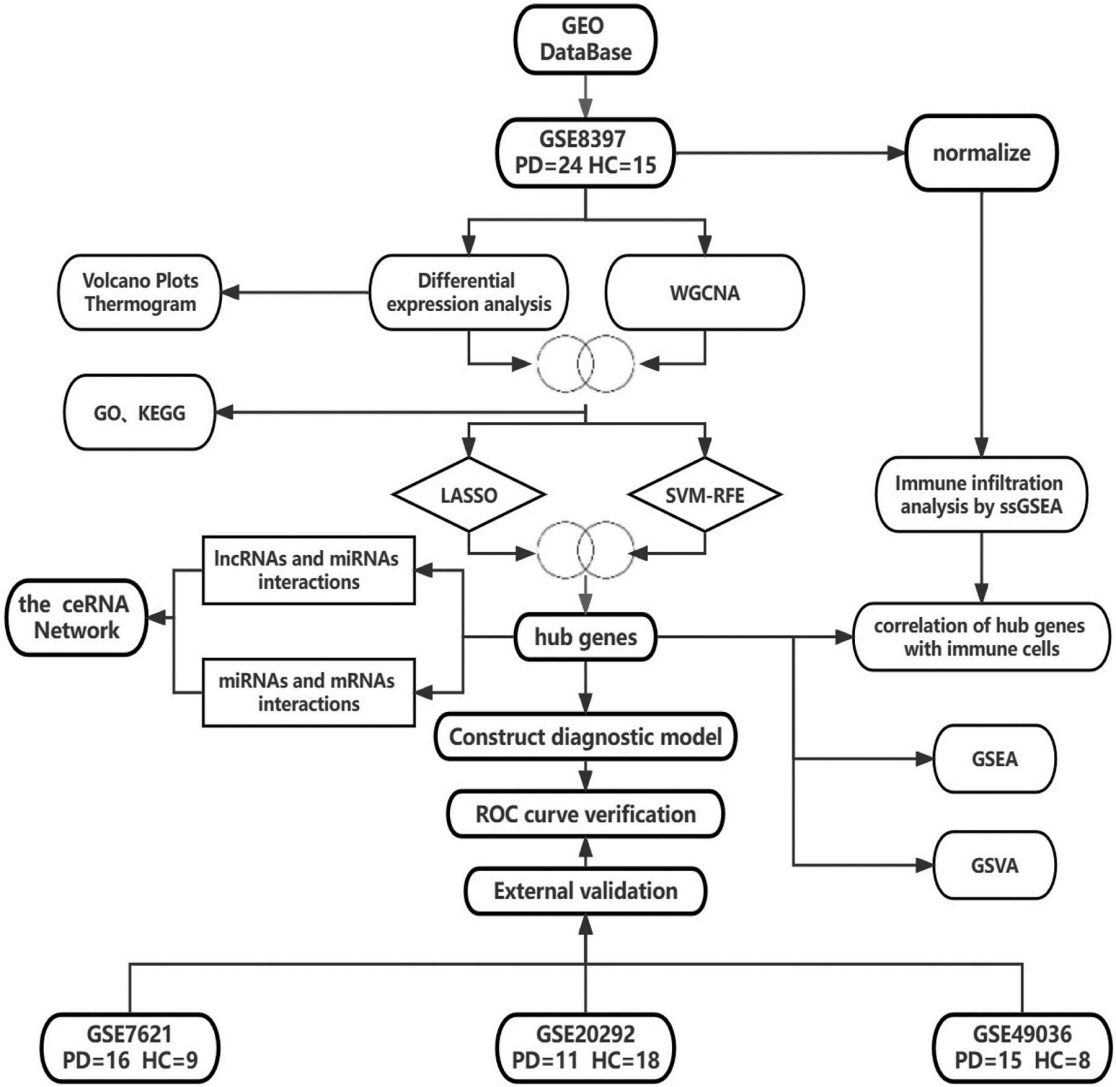
Flowchart of this study. The PD dataset GSE8397 was downloaded from the GEO database. Four Pd hub genes were obtained after processing by differential expression analysis, WGCNA and two machine learning algorithms. A diagnostic model for PD was constructed using these hub genes, and the genes and model were verified to have some accuracy using three independent external datasets. Specific technical details are given in the Methodology section. GEO, Gene Expression Omnibus database; WGCNA, weighted gene correlation network analysis; Lasso, least absolute shrinkage and selection operator; mSVM-RFE (multiple support vector machine-recursive feature elimination); ROC, Receiver Operating Characteristic Curve; GSEA, Gene set enrichment analysis; GSVA, Gene set variation analysis; ceRNA, competitive endogenous RNA; ssGSEA, single-sample Gene Set Enrichment Analysis; GO, Gene Ontology; KEGG, Kyoto Encyclopedia of Genes and Genomes; PD, Parkinson’s disease; HC, Health control; Two circles with overlapping parts mean to take the intersection of two data.

We used weighted gene co-expression network analysis (WGCNA) combined with differential expression analysis to screen for differentially expressed genes (DEGs) between PD and normal control samples. Application of two machine learning algorithms to screen for hub genes and explore potential hub genes associated with immune cells in the substantia nigra of PD patients. A hub gene-based ceRNA regulatory network was constructed, and a logistic regression classification diagnostic model for PD was developed. We present the details of our work in the form of a flowchart to facilitate the reader to quickly understand the details of our work (Fig 1).

## 2. Material and methods

### 2.1 Data preparation

We searched the NCBI GEO (https://www.ncbi.nlm.nih.gov/geo/)database using the keywords "Parkinson’s disease", " Homo sapiens" and " Substantia nigra". Independent datasets with a sample size greater than 20 were selected for the human black matter samples. Datasets with a large number of missing values were excluded. As of September 2022, the GSE8397, GSE7621, GSE20292 and GSE49036 datasets met the above criteria and were included in our study. Specific information on these samples is provided in Table 1. The steps of data processing are divided into two main parts as follows. First, we downloaded the series matrix files of these datasets and downloaded the corresponding platform’s soft annotation list to obtain the microarray probes’ annotation information. In the second part, we used these two files and annotated them based on Perl script to obtain the gene expression matrix file of GSE8397. Finally, we obtained the four datasets’ gene expression matrices and their grouping information. We selected GSE8397, which had the largest number of samples, for bioinformatics analysis. The dataset has 47 samples, of which 15 samples are nigrostriatal tissue from healthy individuals, 24 samples are nigrostriatal tissue from PD patients, and eight samples are post-mortem superior frontal gyrus sources.

**Table 1:** Dataset information from the GEO database.

Location	Accession	Platform	Type	Number
Substantia nigra	GSE8397	GPL96	Microarray	15 control vs. 24 PD
Substantia nigra	GSE49036	GPL570	Microarray	8 control vs. 15 PD
Substantia nigra	GSE20292	GPL96	Microarray	18 control vs. 11 PD
Substantia nigra	GSE7621	GPL570	Microarray	9 control vs. 16 PD

### 2.2 Data preprocessing

**Fig 2:**
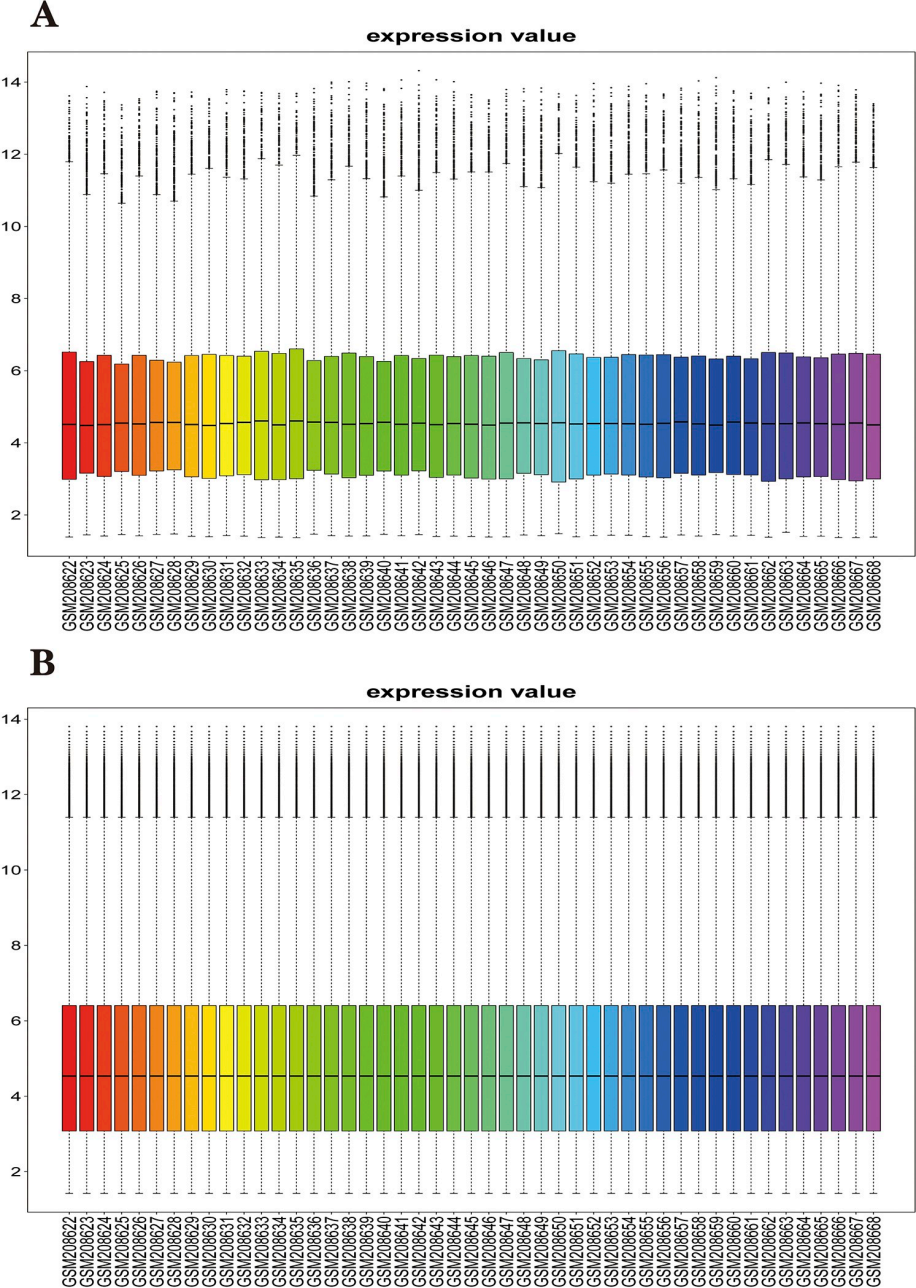
Data normalization. (A) Expression of GSE8397 across samples before data normalization process. (B). Expression of GSE8397 across samples after normalization of data.

We also pre-processed the obtained data before performing the analysis of variance. First, we perform a quality check on the obtained gene expression matrix, including looking at the dimensionality of the data, missing values, outliers, and so on. Second, we plotted box plots to view the distribution of data for each sample in GES8379 and found that the medians of the samples were not at the same level, suggesting the existence of a batch effect across samples due to technical differences. So we normalized the data using the normalizeBetweenArrays function to eliminate technical differences between samples. Plotting the box plot again, we find that the median across samples is on the same straight line, indicating that we have effectively reduced the batch effect (Fig 2).

### 2.3 Differential expression analysis

We used the Limma package (version: 3.52.2) in R software to perform differential expression analysis on the expression profile dataset of GSE8397 [16]. The significance criterion for Differential expression genes (DEGs) was set at FoldChang>1.2 and an adjusted P value of <0.05. Heat map of DEGs with the pheatmap package (version 1.0.12). The volcano map drawn with the ggplot2 package (version 3.3.6) indicates differentially expressed genes. Unless otherwise stated, the version of R software used in this study is version 4.2.1.

### 2.4 Construction of weighted gene co-expression network

We analyzed GSE8397 using the “WGCNA” package in R software to explore the gene expression and interactions in PD samples [17, 18]. First, we calculated the standard deviation of these genes and excluded genes with standard deviations <0.5. A scale-free co-expression network was constructed by calculating the Pearson correlation coefficient between every two genes. We use the hclust function to cluster the samples to determine the modules. The Pick-Soft-Threshold function is then used to calculate the adjacency values, retaining the soft threshold based on co-expression similarity. Second, the adjacency values are converted into a topological overlap matrix (TOM) to measure the average network connectivity of each gene. The dissimilarity between genes (1-TOM) was calculated. Third, hierarchical clustering of topological overlap matrices was performed using an averaging algorithm to identify clusters of interrelated genes. We limit the minimum number of genes per module to 30, with a depth partition of 2. We use a dynamic tree-cutting method to divide genes with similar expression profiles into different modules. Fourth, a threshold of 0.25 is used to merge the strongly correlated modules to obtain the correlated modules. Calculate the module eigengene (ME) value for each module. In addition, correlation coefficients and p-values between ME values and clinical trait phenotypes were calculated. Heat maps of correlations between modules and sample traits were drawn. We selected the brown module with the highest correlation to the healthy group (Normal group) and the PD group for further analysis. Genes with gene significance (GS) > 0.4 and module membership (MM) > 0.8 were defined as module key genes.

### 2.5 Functional enrichment

We obtained 350 differentially expressed genes after taking the intersection of module key genes with DEGs. The results were visualized using Venn diagrams [19]. Hiplot (https://hiplot-academic.com/) was used to analyze the potential functions of these differentially expressed genes. These analyses include Gene ontology (GO) and Kyoto Encyclopedia of Genes and Genomes (KEGG) analysis [20, 21]. The specific parameters are setting the minimum gene set to 10 and the maximum gene set to 500. The database used is public/cd/kegg/hsa_kegg_20210213.rds. Statistically significant pathways were identified as p-values <0.05 and q-values <0.1

### 2.6 Screening for hub genes by LASSO and mSVM-RFE

We use two machine learning algorithms, Least Absolute Shrinkage and Selection Operator (LASSO) and multiple Support Vector Machine Recursive Feature Elimination Algorithm (mSVM-RFE), to identify the most relevant hub genes for PD.

LASSO employs a penalty term that encourages some model coefficients to be exactly zero, effectively selecting a subset of the most relevant features while shrinking the others towards zero. By using LASSO to identify the most informative features while reducing the risk of overfitting. The methodology is as follows: we use the glmnet package (version: 4.1–4) to build the LASSO models [22, 23]. The specific parameter settings were: response type was set to binomial, alpha was set to 1, and 10-fold cross-validation was performed to adjust the optimal value of the parameter λ.

SVM-RFE is a machine-learning algorithm that finds the best variables by removing the feature vectors generated by the SVM. However, this algorithm is only applicable to the binary case and has a higher error rate when faced with the multiclassification case. mSVM-RFE resembles the steps of SVM-RFE, however, at each step a ranking score is computed once, which is derived from the statistical computation of the coefficients of the weight vector. We modeled the mSVM-RFE using the e1071 package (version: 1.7–11) [24]. The parameters are set as follows: halfve.above = 50. Using 10-fold cross-validation, the average feature gene rankings of the 10 training sets were obtained, after which the average generalization error of the feature genes was plotted against the number of features, and the number of feature genes with the minimum error rate was found after comparison to make the algorithm more accurate.

Our main optimization goal for the mSVM-RFE model is to minimize false positives, while the LASSO model is used for feature selection and data dimensionality reduction. In addition, the intersection of the genes obtained by the two algorithms was taken to determine hub genes for PD [25].

### 2.7 Gene Set Enrichment Analysis (GSEA)

GSEA can be used to elucidate whether a set of genomes is significantly different in two biological states [26]. To further explore the potential mechanisms by which these four hub genes affect PD, we performed a GSEA analysis for this purpose [27]. In the GSE8397 dataset, their correlation coefficients with other genes were calculated separately by spearman analysis according to the expression levels of the hub genes. Organize the correlation coefficients into a list of input genes for the clusterProfiler package (version 4.4.4). Meanwhile, "c2.cp.kegg.v7.5.1.symbols.gmt" was downloaded from the MSigDB database as a reference gene set to check its abundance in the gene collection.

### 2.8 Gene Set Variation Analysis (GSVA)

GSVA is a non-parametric and unsupervised method for assessing the enrichment of transcriptomic gene sets [28]. We used the GSVA package of the R program to calculate and score the genes obtained from machine learning [29]. Also, the limma package of the R program was used to calculate the difference in scores between overexpressed and below-normal expression samples of genes obtained from machine learning. The version of our reference gene set is "c2.cp.kegg.v7.5.1.symbols.gmt". Statistically, significant paths were determined as p-value < 0.05.

### 2.9 Correlation analysis between hub genes and immune characteristics

To determine the role of hub genes in the immune microenvironment, we applied the single-sample Gene Set Enrichment Analysis (ssGSEA) algorithm to calculate the distribution of 28 immune cells in 39 samples associated with PD nigra in the dataset GSE8397 [30]. The correlation between these samples and immune cell infiltration was analyzed. The correlation analysis was visually transformed using the pheatmap package of R software and the vioplot package. The GSVA (version: 3.4.2) package was used as a tool for ssGSEA analysis [26].

### 2.10 Construction of competing endogenous RNAs (ceRNA) network

First, three public databases, namely miRanda, miRDB and TargetScan, were used to simultaneously predict the target miRNA of the Parkinson’s disease Hub gene, and the prediction results of the three databases were intersected to obtain the final mRNA-miRN mutual correspondence result. Secondly, using the mRNA-miRN correspondence results obtained in the previous step, the target lncRNA was predicted through the SpongeScan public database, and the miRNA-lncRNA correspondence results were obtained. Finally, based on the results obtained in the above two steps, the ceRNA regulatory network was constructed. The ceRNA regulatory network was visualized using Cytoscape software.

All data in this study were obtained from information published in the GEO public database. GSE8397 data as the study subject were collected from September 2007, and we analyzed these data in September 2022; GSE49036, GSE20292, and GSE7621 data as the validation set were collected from June 2013, February 2010, and April 2007, respectively.

## 3. Result

### 3.1 Differential expression analysis

**Fig 3:**
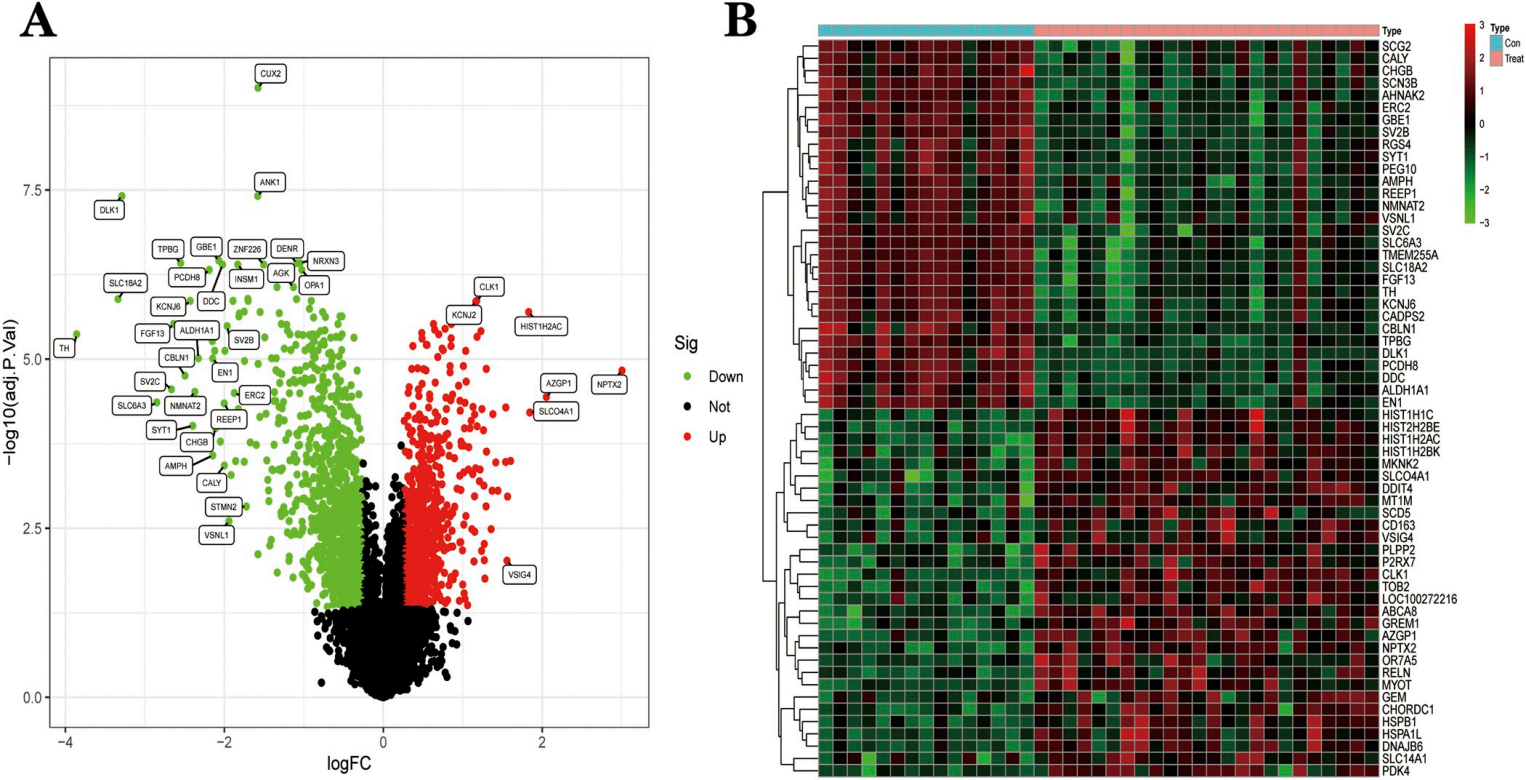
**(A)** The differential expression analysis results are shown in the volcano plot. Where the x-axis represents log2 (fold change) and the y-axis represents -log10 (adjust p. value). Green dots represent downregulated genes, red dots represent upregulated genes, and black dots represent genes with no obvious differential expression. **(B)** Heatmap of the top 60 differentially expressed genes. Each column in the graph represents a sample, each row represents a gene, and the expression status of the genes is indicated from high to low in red to green, respectively, and at the top of the heat map, blue/red represents the Health group/ PD group.

We calculate the differences in gene expression between patients in the PD group and those in the healthy group. Finally, we obtained 2086 genes (DEGs) that were differentially expressed compared to the healthy group. This includes 1141 genes that are lowly expressed relative to the healthy group. 945 genes were highly expressed relative to the healthy group. We visualized the results using a volcano map (Fig 3A) and a heat map (Fig 3B). Details of the calculation are given in S1 Appendix.

### 3.2 Weighted gene correlation network analysis

**Fig 4:**
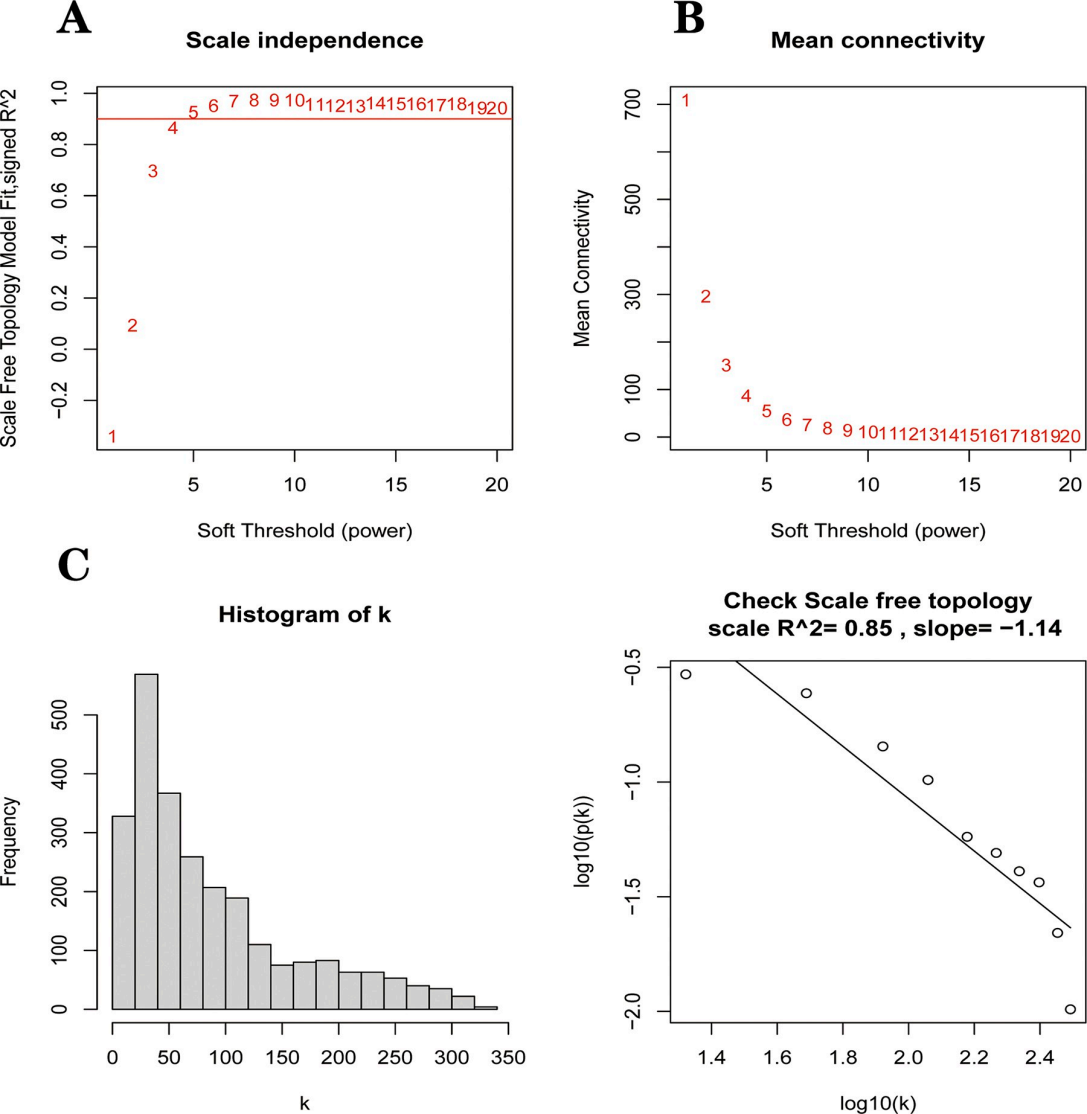
Data quality control before constructing WGCNA. **(A)**Scale-free index for analyzing the power of various soft thresholds. The horizontal coordinate represents the power of soft thresholds, and the best soft threshold is marked with a red line. **(B)** Average connectivity of various soft thresholds. **(C)** Check whether the set soft threshold satisfies the scale-free network.

**Fig 5:**
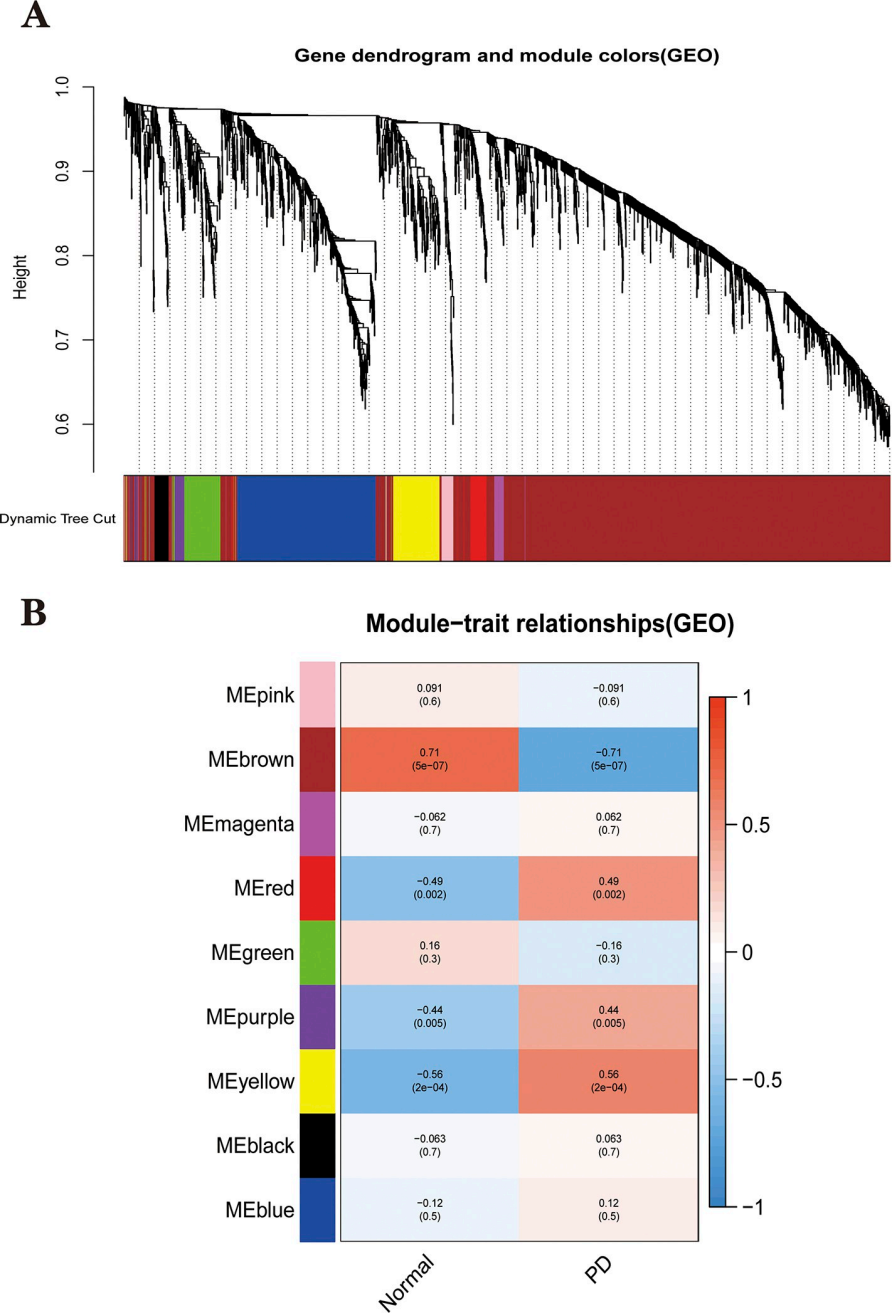
Utilizing WGCNA to obtain key modules. **(A)**Identification of co-expressed gene modules. A dendrogram of all differentially expressed genes was clustered based on a measure of gene similarity. Cut lines of modules were identified, and a different color indicated each module. **(B)** Heat map of the correlation between modules and clinical phenotypes. module and the clinical phenotype. The corresponding cor value and p-value are labeled therein. The brown modules have the strongest correlation with PD.

**Fig 6:**
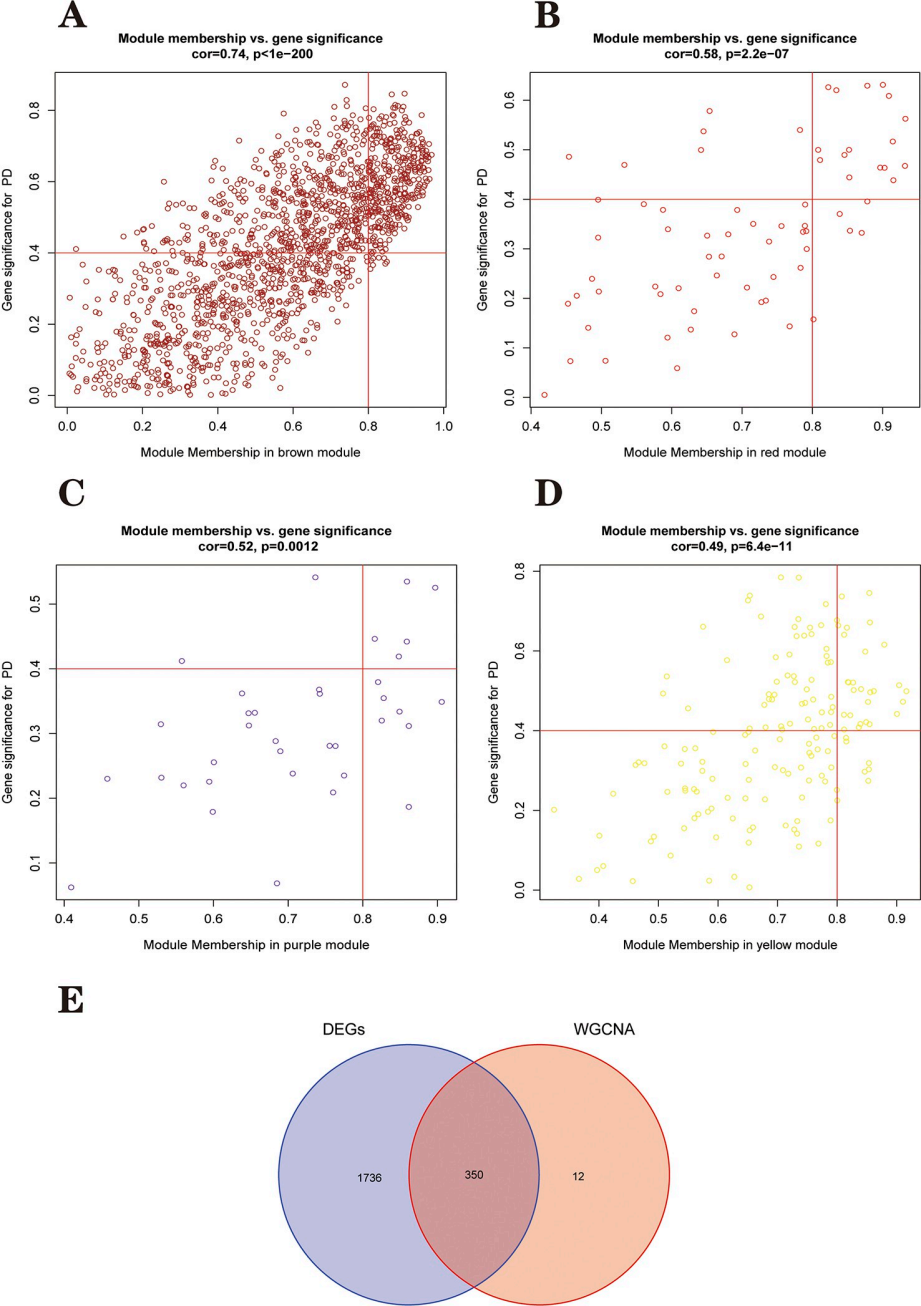
Correlation of Module Membership (MM) with Gene Significance (GS) in the four most relevant modules for PD. **(A)** Scatterplot of brown modules. **(B)** Scatterplot of red modules. **(C)** Scatterplot of purple modules. **(D)** Scatterplot of yellow modules. **(E)** A total of 350 DEGs were screened for further analysis. Red represents 362 genes in the module with the strongest correlation to the PD group, blue represents the 2086 genes obtained from differential expression analysis.

A total of 24 PD samples, 15 healthy control samples, and 13101 genes were included in the WGCNA analysis. Genes with standard deviation <0.5 and deleted values were removed. Cluster analysis of all samples showed no outliers. Ultimately, 2547 genes from 39 samples were used to construct a weighted gene co-expression network. After calculation, the optimal soft threshold was determined as 4 (Fig 4A and 4B). To check whether the optimal soft threshold satisfies the scale-free network, we plotted log10(p(k)) and log10(k). We calculated the square of the log(k) and log(p(k)) correlation coefficients corresponding to different power values, i.e., R2. If the R2 of the model is close to 1, the relationship is well linear. The higher the value, the more the gene association fits the scale-free distribution. Fig 4C shows that the R2 value for our constructed model is 0.85. Subsequently, we transformed this adjacency matrix into a TOM matrix (topological overlap matrix), which better reflects the connectivity and adjacency relationships between genes. The minimum number of genes in the module was set to 30, and modules with fewer genes than this value were merged into larger modules, setting the shear height to 0.25, and we ended up with nine modules (Fig 5A). Heat maps of the modules and clinical traits were drawn (Fig 5B). Gray modules are used to place genes that are not attributed to any of the modules and are not considered to be of clinical analysis. Correlations between model eigengenes (ME) and clinical characteristics were calculated using Pearson correlation coefficients for each module, including Brown, Red, Purple and Yellow for the four modules at P<0.05 [31]. We then calculated the relationship between gene expression levels and clinical performance for these four modules using Pearson correlation coefficients. We plot the scatter plots of GS and MM correlations for the four modules in Brown, Red, Purple and Yellow (Fig 6A–6D). Combining Fig 5B and Fig 6A–6D we can intuitively find that the brown module (cor = -0.74, p<1e-200) has the highest clinical correlation with the PD group. We extracted 362 module key genes from the brown module based on the threshold of GS>0.4 and MM>0.8(S2 Appendix).

### 3.3 Functional analyses

**Fig 7:**
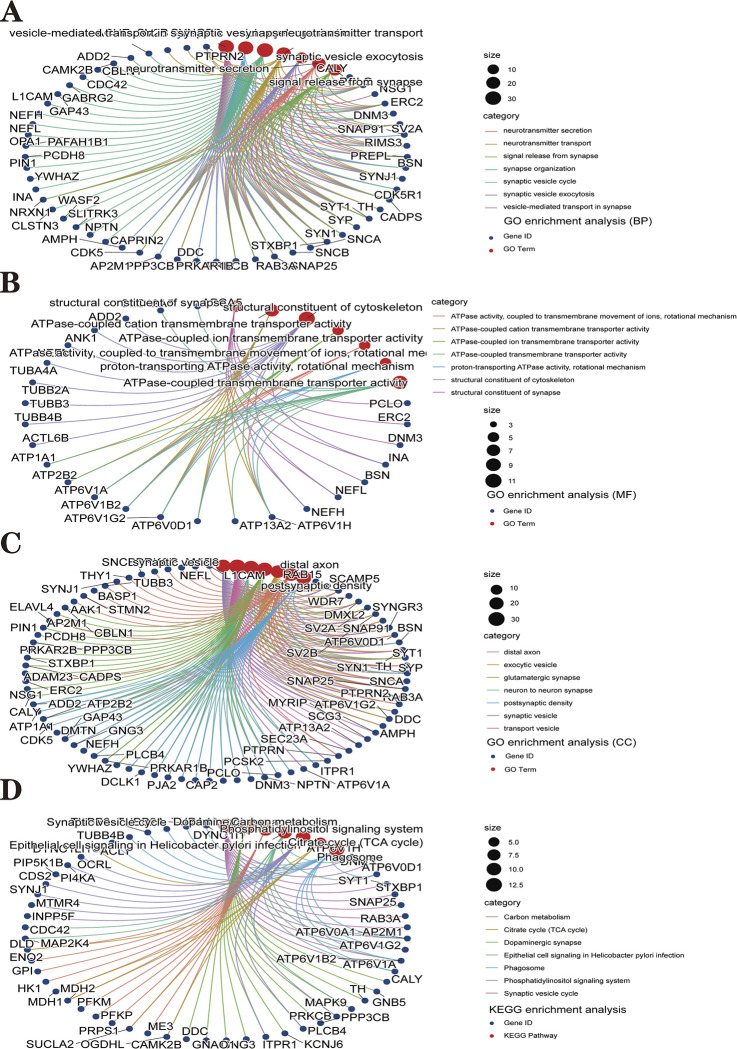
The GO and KEGG enrichment analysis results of 350 DEGs are shown as circle plots. **(A)** Shows the top 7 significantly enriched BP (biological process). **(B)** Shows the top 7 enriched CC (cellular component) considerably. **(C)** The top 7 enriched MF considerably (molecular function) are shown. **(D)** The top 7 significantly enriched KEGG pathways. In the circle plot of GO and KEGG enrichment analysis, the size of the dots represents the number of genes.

We obtained 350 differentially expressed genes after taking the intersection of module key genes and DEGs. Visualize the results using Venn diagrams (Fig 6E). We performed GO enrichment analysis and KEGG pathway analysis on these differentially expressed genes to elucidate the biological functions and pathways associated with PD. GO enrichment analysis showed that these differentially expressed genes were significantly associated with function in vesicles, neurotransmitters, ATPase activity, structural constituent of the cytoskeleton, and glutamatergic synapses (Fig 7A–7C). KEGG pathway analysis showed that differentially expressed genes were enriched in the synaptic vesicle cycle, dopaminergic synapses, phagosomes, carbon metabolism, phosphatidylinositol signaling system, and Citrate cycle (TCA cycle) pathways (Fig 7D). The specific enrichment results of DEGs are integrated into S3 Appendix.

### 3.4 Two machine learning approaches to identify hub genes

**Fig 8:**
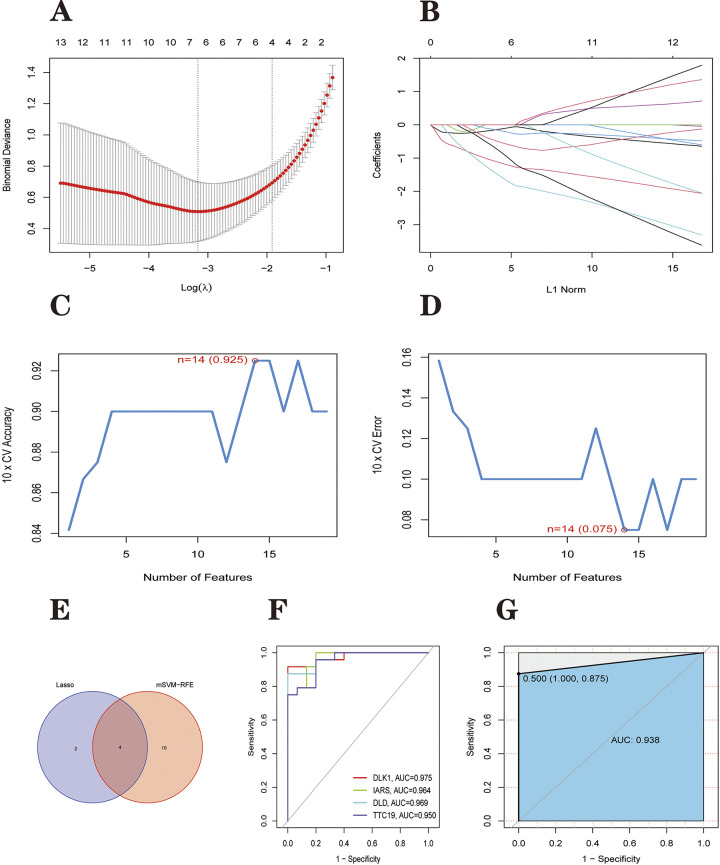
4 genes were identified as hub genes for PD. (A and B) The LASSO logistic regression algorithm selected six PD-related features. (C and D) mSVM-RFE algorithm to filter DEGs to identify the best combination of signature genes. Finally, 14 genes were identified as the best-characterized genes. (E) hub genes were obtained from the LASSO and mSVM-RFE models. (F) ROC curve to test the diagnostic capability of the logistic regression model. (G) ROC curves to test the diagnostic ability of 4 hub genes. The maximum value of the area under the ROC curve was 0.938, corresponding to a cut-off point of 0.500, a specificity of 1.000 and a sensitivity of 0.875.

LASSO logistic regression algorithm with penalized parameter adjustment by 10-fold cross-validation to select six features associated with PD (Fig 8A and 8B). Optimal lambda.min value = 0.04205185. At the same time the lambda.1se value of the model is 0.1476521. We built the mSVM-RFE model for feature ranking. k = 10 means it is ten times cross-validation. alive.over = 100 means that the features are halved each round of the cycle, i.e., the number of features is halved each round until the number of features (number of genes) left is less than 100. The output is a vector of feature indices, i.e., a ranking from "most useful" to "least useful". After that, the average false positive rates of 10 cross-validations were compared. Finally, 14 genes (maximum accuracy = 0.925, minimum root mean square error = 0.075) were identified as the best-characterized genes (Fig 8C and 8D). Marker genes obtained from the LASSO and mSVM-RFE models were crossed, and four central genes (dlk1, iars, dld and ttc19) were identified for subsequent analysis (Fig 8E). We randomly divided GSE8397 into 70% as the training set and 30% as the internal validation set for model learning and validation. ROC curves were generated for four hub genes to elucidate the ability of individual genes to distinguish PD from normal samples. As shown in Fig 8F, the area under curve (AUC) values of all genes were greater than 0.900 (*dlk*1 = 0.975, *iars* = 0.964, *dld* = 0.969 and *ttc*19 = 0.950), indicating that all four genes had good diagnostic ability. We constructed a logistic regression classification diagnostic model using these four genes. The ROC curve showed the diagnostic model with the area under the curve (AUC) = 0.938, indicating that the logistic regression classification diagnostic model based on four hub genes has good classification ability (Fig 8G). But the point that must be stated is that the reason for such high AUC values for these four genes may be that the genes were selected based on the same sample. So we subsequently used three independent external datasets to verify the accuracy of these four hub genes.

### 3.5 GSEA and GSVA enrichment analysis

**Fig 9:**
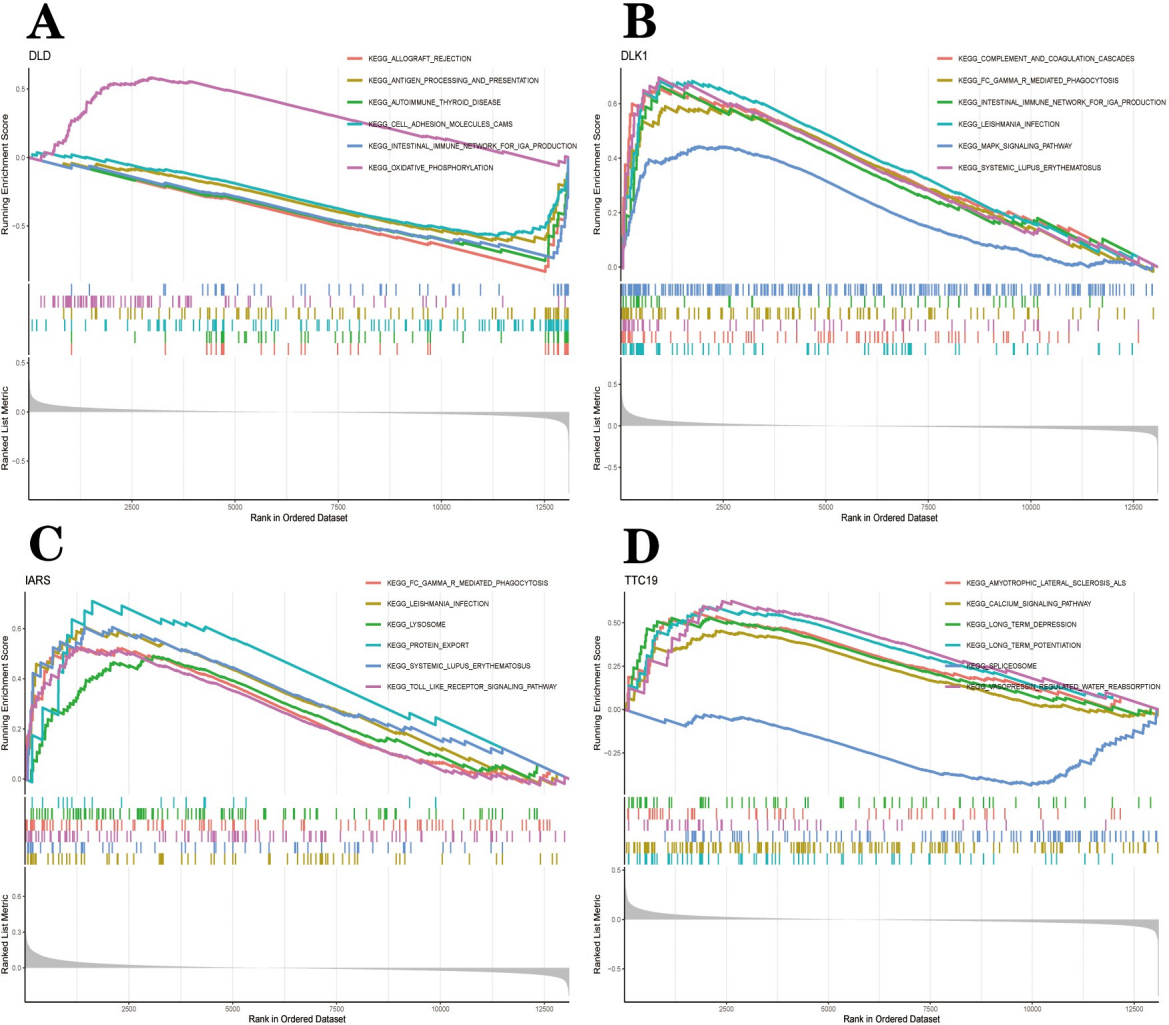
GSEA- KEGG pathway analysis in *dld* (A), *dlk*1 (B), *iars* (C) and *ttc*19(D).

**Fig 10:**
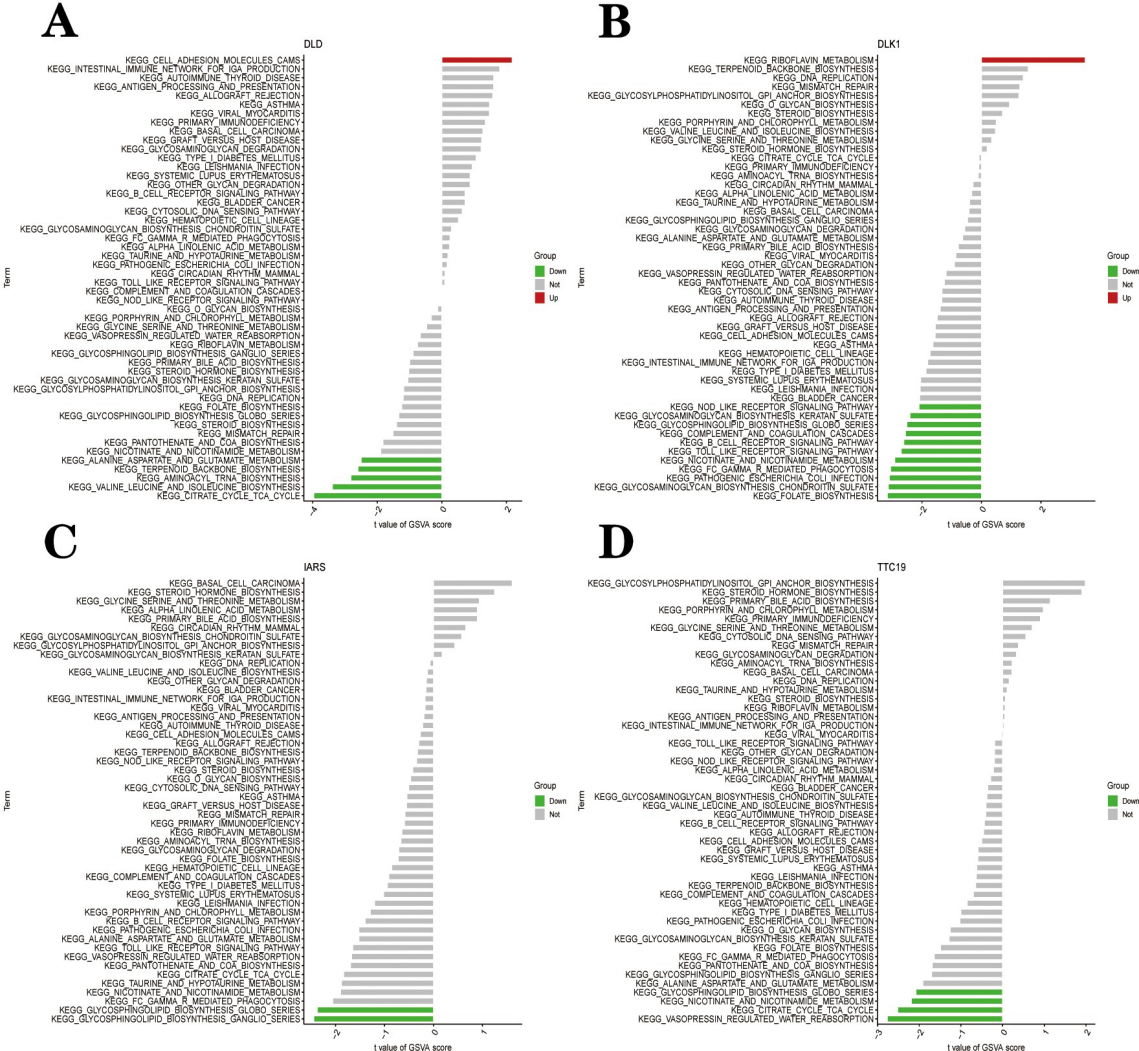
High and low-expression groups based on the expression levels of each marker gene combined with GSVA in *dld* (A), *dlk* 1 (B), *iars* (C) and *ttc*19(D).

To further explore the potential function of the hub gene to distinguish disease samples from normal samples, we performed a single-gene GSEA-KEGG pathway analysis. Fig 9A–9D shows the first six pathways for each marker gene enrichment. After a comprehensive analysis, we found hub genes in the lysosome, oxidative phosphorylation, spliceosomes, vasopressin-regulated water reabsorption, leishmaniasis infection, immune response (complement and coagulation cascade, graft versus host disease, Antigen processing and expression, Fc gamma R mediated phagocytosis, the intestinal immune network for gor IgA production, cell adhesion molecule CAMS) and various immune-related disease pathways (autoimmune thyroid disease, amyotrophic lateral sclerosis ALS, systemic lupus erythematosus SLE). In addition, we found that these central genes were also enriched in the MAPK signaling pathway, Toll-like receptor signaling pathway and calcium signaling pathway. The specific enrichment results for each gene are integrated into S4 Appendix. We then combined GSVA to look at the different activation pathways between the high and low-expression groups based on the expression level of each marker gene (Fig 10A–10D). As shown in the figure, overexpression of *dld* can induce PD by activating the CAM pathway, a cell adhesion molecule. Low expression of *dld* can cause PD by association with multiple biosynthetic and TCA cycle pathways. Upregulation of *dlk*1 activates the riboflavin metabolic pathway, which induces PD. Downregulation of *dlk*1 activates pathways of folate biosynthesis, pathogenic E. coli infection, Toll-like receptor signaling pathways and immune-related signaling pathways to cause PD. *iars* induces PD through downregulation of the Glycosphingolipid biosynthesis globo series and Glycosphingolipid biosynthesis ganglio series. *ttc*19 generates PD through the downregulation of nicotinic acid and nicotinamide metabolism, citric acid cycle TCA cycle and Vasopressin regulation of water reabsorption only.

### 3.6 Correlation analysis between hub genes and immune characteristics

**Fig 11:**
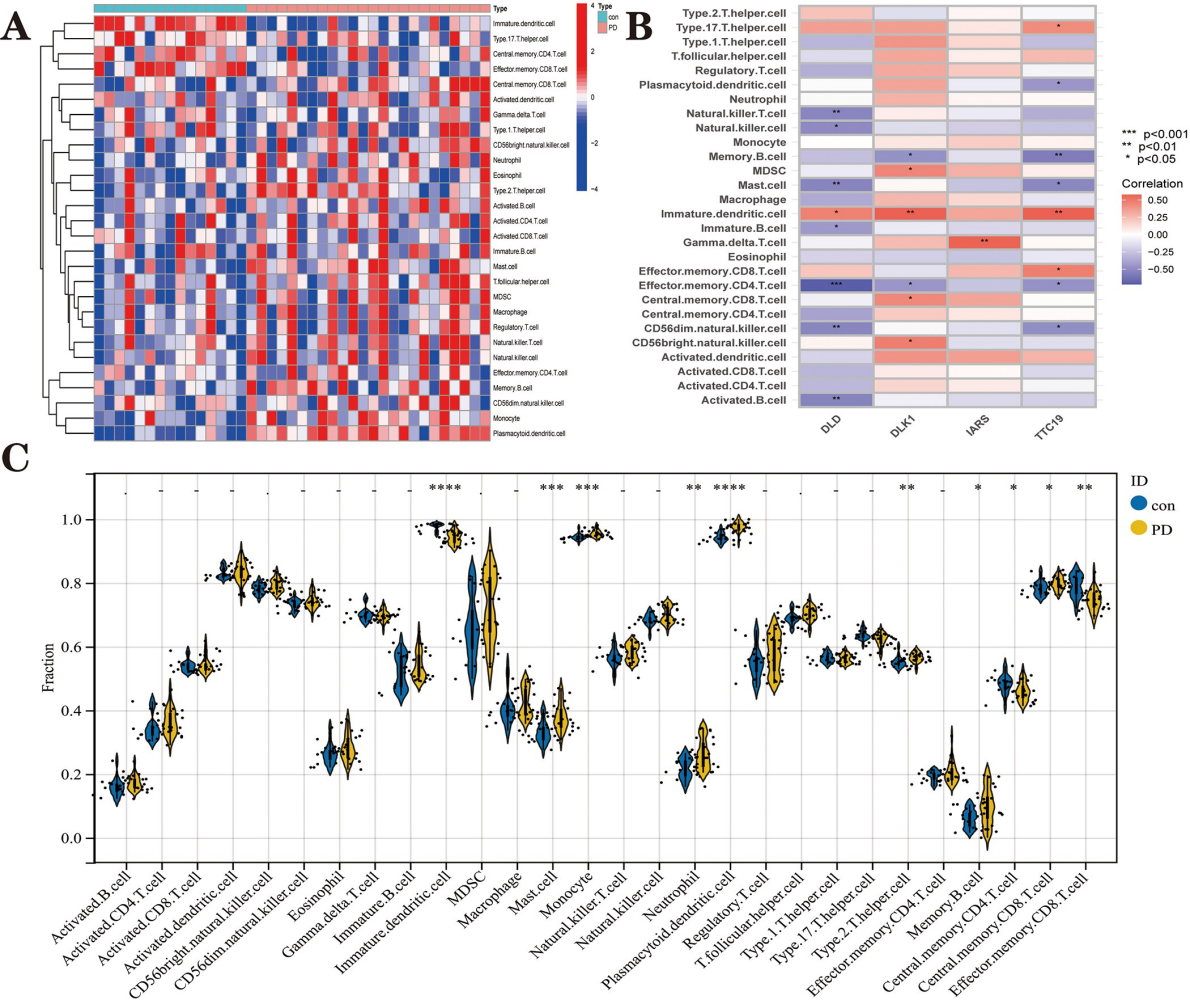
Immune infiltration landscape between PD sample and CON sample (health sample) obtained by ssGSEA analysis. **(A)** Heat map summarizing the scores of immune cell infiltration between PD patients and non-PD patients. **(B)** Violin plot showing the difference in immune cell infiltration between PD (yellow) and CON (blue), p < 0.05, was considered statistically significant. The more asterisks in the violin, the stronger the correlation. **(C)** Shows the correlation between hub genes and immune cells. The colors from brown to green represent the change from positive to negative correlations, respectively. More asterisks and darker colors of the modules represent stronger correlations.

We used the ssGSEA method to quantify the immune infiltration fraction of 28 immune cells in the samples and the immune infiltration heat map (Fig 11A and S5 Appendix) to explore the differences in the immune microenvironment between PD patients and healthy samples. The proportions of Immature dendritic cells, Effector memory CD8 T cells and central memory CD4 T cells were significantly lower in PD patients than in patients in the healthy group (Fig 11C). Mast cells, monocytes, neutrophil, Plasmacytoid dendritic cell, type 2 T helper cells, memory B cells and central memory CD8 T cells were significantly higher in the PD group than in the healthy group. The results showed (Fig 11B) that Immature dendritic cell was positively correlated with *dld*, *dlk*1 and *ttc*19, and Effector memory CD4 T cell was negatively correlated with *dld*, *dlk*1 and *ttc*19. In addition, *dld* was negatively correlated with CD56dim nature killer cell, Master cell and Nature killer T cell. *dlk*1 was positively correlated with CD56bright nature killer cell. *ttc*19 was negatively correlated with the Memory B cell. *iars* has positively correlated with Gamma delta T cells. This evidence suggests that hub genes may play a role in the development of PD by regulating the immune microenvironment.

### 3.7 Construction of lncRNA–miRNA–mRNA competitive network

**Fig 12:**
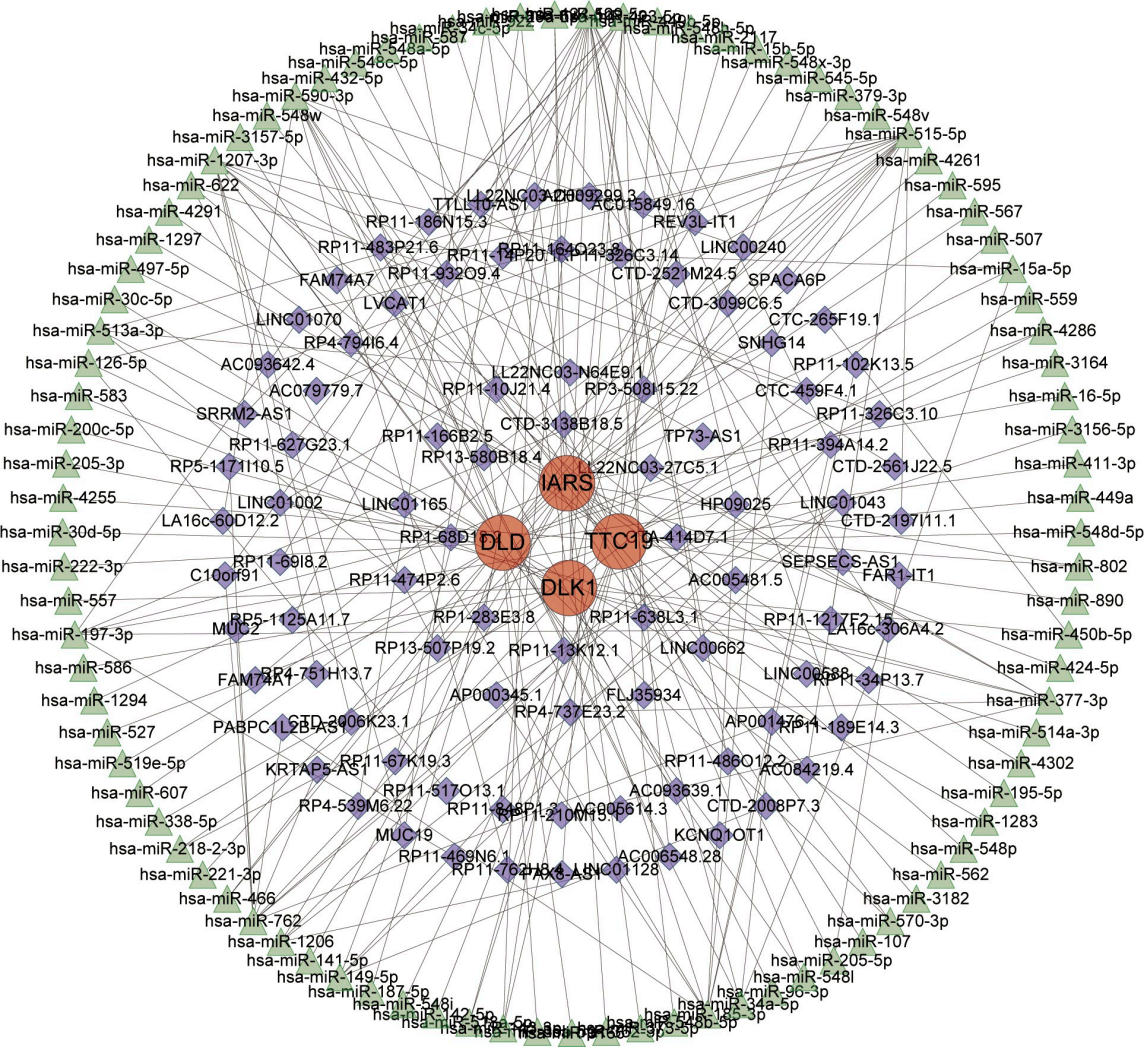
A ceRNA networks based on hub genes. The network included 188 nodes (4 hub genes, 93 miRNAs and 91 lncRNAs) and 207 edges.

The network includes 188 nodes (4 hub genes, 93 miRNAs and 91 lncRNAs) and 207 edges (Fig 12). The specific details of the ceRNA network are shown in S6 Appendix. According to Degree analysis, miRNAs such as hsa-miR-515-5p, hsa-miR-423-5p, hsa-miR-129-5p, hsa-miR-590-3p, hsa-miR-1207-3p, and hsa-miR-377-3p may play important regulatory roles in the network. SNHG14, HP09025, LINC01043, LA16c-306A4.2 and RP11-13K12.1 are lncRNAs that may play important regulatory roles in the network.

### 3.8 Validation of hub genes and diagnostic models

**Fig 13:**
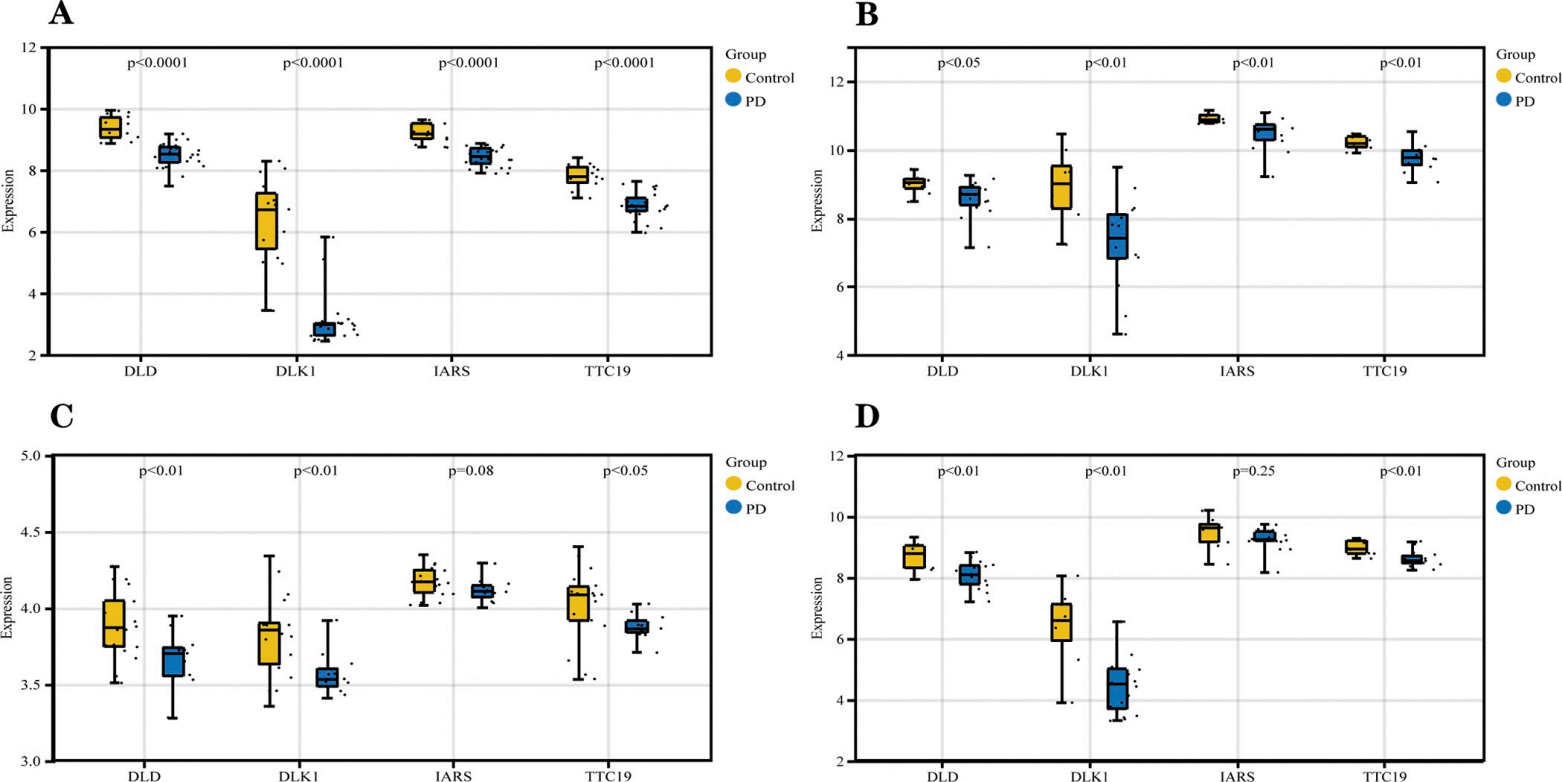
Statistical analysis of hub genes’ parameters in different datasets. (A) Parametric statistical analysis of Hub genes in GSE8397. (B) Parametric statistical analysis of Hub genes in GSE49036. (C) Parametric statistical analysis of Hub genes in GSE20292.(D) Parametric statistical analysis of Hub genes in GSE7621. P < 0.05 was considered statistically different.

**Fig 14:**
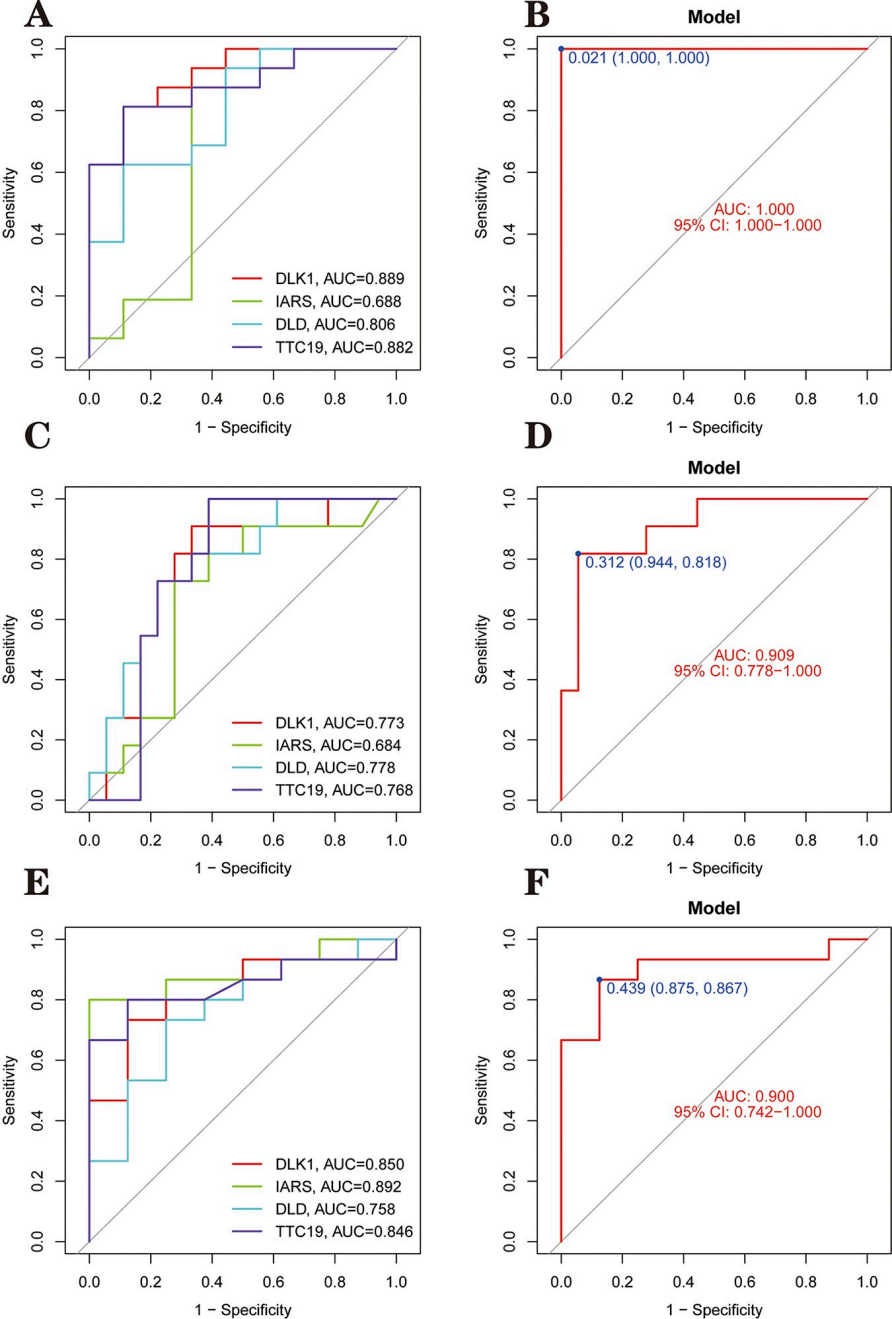
Validation of hub genes and diagnostic models. **(A&B)** ROC curves for the validation set GSE7621 dataset. The maximum value of the area under the ROC curve was 1.000, corresponding to a cut-off point of 0.021, a specificity of 1.000 and a sensitivity of 1.000. **(C&D)** ROC curves for the validation set GSE20292 dataset. The maximum value of the area under the ROC curve was 0.909, corresponding to a cut-off point of 0.312, a specificity of 0.944 and a sensitivity of 0.818. **(E&F)** ROC curves for the validation set GSE49036 dataset. The maximum value of the area under the ROC curve was 0.900, corresponding to a cut-off point of 0.439, a specificity of 0.875 and a sensitivity of 0.867. The different colored lines in the left graph represent different genes, and the right graph indicates the ROC curve of the diagnostic model.

Before validation, we also used parametric tests to analyze whether the four genes in each dataset differed significantly between PD and healthy individuals. The specific statistical method was the t-test. It should be noted that we have normalized GSE8397 and the three independent validation datasets using the normalizeBetweenArrays function during the data preprocessing stage before proceeding with data analysis. Ultimately, we found that the iars gene did not differ between groups in the GSE7621 and GSE20292 datasets (Fig 13). After that, we use three independent external datasets (GSE7621, GSE20292 and GSE49036) to validate the accuracy of our established model. One point to note is that although we trained the classification model on the GSE8397 dataset by randomly dividing the dataset according to 70/30, we did not randomly divide the external independent dataset during validation. This approach was designed to test the performance of the model on real-world data to simulate how the model would be used in clinical applications. In validating the model’s accuracy using an external independent dataset, we calculated the AUC values of the four hub genes and the AUC values of the model. We found that the AUC values for the four genes in GSE7621 were *dlk*1 = 0.889, *iars* = 0.688, *dld* = 0.806 and *ttc*19 = 0.882, while the combined diagnostic model had an AUC = 1.000 (Fig 14A and 14B). In GSE20292, the AUC values for the four genes were *dlk*1 = 0.773, *iars* = 0.684, *dld* = 0.778 and *ttc*19 = 0.768 and AUC = 0.909 for the combined diagnostic model (Fig 14C and 14D). In GSE49036, the AUC values for the four genes were *dlk*1 = 0.850, *iars* = 0.892, *dld* = 0.758 and *ttc*19 = 0.846, and the AUC for the combined diagnostic model = 0.900 (Fig 14E and 14F). It can be seen that *dlk*1, *dld* and *ttc*19 have AUC values greater than 0.7 in three external independent datasets, indicating that these three genes have a certain level of accuracy. The *iars* gene had AUC values between 0.5 and 0.7 in two external independent datasets and between 0.7 and 0.9 in one external independent dataset, indicating that this has some research value. The AUC values of the combined diagnostic model in the four independent data sets were greater than 0.900, indicating that the combined diagnostic model had good classification performance for PD samples and normal samples.

## 4. Discussion

In recent years, the incidence of PD has been increasing as the average life expectancy of humans has increased. The number of people with PD worldwide is expected to be twice as high by 2030 as it was in 2016. Considering that current treatments for PD are limited to improving symptoms in the early stages of the disease and lack etiologic treatments, there is an urgent need to develop effective therapeutic options to alleviate the increasing burden of the disease on patients and their families. In recent years, the use of bioinformatics methods to explore key disease-causing genes and potential drug targets has become a new hot spot within the field of life sciences. More and more studies indicate that immunity may play some kind of key role in neurodegenerative diseases, and it is hoped that new therapeutic targets related to PD immunity will be unearthed to enrich the pathogenic mechanisms and intervention pathways of PD, and then realize the ultimate goal of eradicating PD. In addition, the development of new biomarkers of the disease provides a research basis for early detection and early intervention in Parkinson’s disease.

In this study, we combined WGCNA and differential expression analysis to obtain differentially expressed genes (DEGs) in PD nigrostriatal tissue and performed GO and KEGG enrichment analysis. GO analysis showed that DEGs were significantly enriched in vesicles, neurotransmitters, ATPase activity, structural constituent of the cytoskeleton and glutamate synaptic functions. KEGG pathway analysis revealed that DEGs were enriched in the synaptic vesicle cycle, dopaminergic synapses, phagosomes, carbon metabolism, phosphatidylinositol signaling system and citric acid cycle (TCA cycle) pathways. Previous studies have demonstrated that vesicular transport and lysosomal dysfunction impair the clearance of α-synuclein [32, 33]. The role of carbon metabolism and the TCA cycle in PD has also been gradually discovered, and these studies deserve further exploration [34, 35].

Then four hub genes: *dlk*1, *iars*, *dld* and *ttc*19 were obtained by two machine learning algorithms, LASSO and mSVM-RFE. The *dld* and *dlk*1 genes were considered potential biomarkers of PD in previous studies [36, 37].

Altered energy metabolism, including reduced activity of the key mitochondrial enzymes α-ketoglutarate dehydrogenase complex (KGDHC) and pyruvate dehydrogenase complex (PDHC), characterizes many neurodegenerative diseases such as Alzheimer’s disease (AD) and Parkinson’s disease [38, 39]. The *dld* genes encode dihydroacetylamine dehydrogenase, a mitochondrial protein that is a key subunit of KGDHC and PDHC and plays an important role in energy metabolism [40]. Klivenyi and Schmidt et al. found an increased sensitivity of *dld*-deficient mice to 1-methyl-4-phenyl-1,2,3,6-tetrahydropyridine (MPTP), malonic acid and 3-nitropropionic acid (3-NP), which are important tools for the construction of Parkinson’s disease models [41, 42]. GSVA analysis also showed that *dld* was mainly enriched in pathways related to energy metabolism and species metabolism. In contrast, GSEA analysis showed that *dld* was enriched primarily on immunity-related pathways. Years of research have long judged the importance of energy metabolism for PD, and studies targeting upstream and downstream of dld may bring new insights into the pathogenesis of PD.

The *dlk*1 gene encodes Delta Like Non-Canonical Notch Ligand 1. The *dlk*1 gene belongs to the epidermal growth factor superfamily and is considered a ligand for the Notch receptor. The interaction between Notch receptors and their ligands is critical for the development of various tissues. Jacobs et al. found that *dlk*1 plays an important role in the development of midbrain dopamine neurons, especially by preventing the premature expression of dopamine transporter proteins, which helps to maintain the normal function of dopamine neurons. In addition, *dlk*1 is involved in neuronal differentiation and Parkinson’s disease pathology along with other genes such as Nurr1 and Pitx3 [43]. The *dlk*1 gene has been suggested as a potential complementary marker of dopaminergic neurons. Liechti et al. found that *dlk*1 expression was significantly increased in cells positive for a key enzyme (tyrosine hydroxylase) that synthesizes dopamine during postnatal developmental stages in mice. In contrast, a significant increase in the number of dlk1-immunoreactive(-ir) cells relative to unimpaired controls was detected in dopamine-depleted striatum [44]. This suggests that *dlk*1 may be involved in the cellular response to degenerative processes. This does not rule out a protective response by the organism. GSEA analysis showed that this gene is mainly enriched in immune-related pathways and the MPKA signaling pathway, which regulates various cellular activities including proliferation, differentiation, survival and death. GSVA analysis showed that *dlk*1 is associated with immune-related and synthetic pathways of genetic material. We expect that future studies will explore whether this gene is somehow associated with the course of PD, and then try to detect the gene in peripheral blood.

The *iars* gene encodes Isoleucyl-TRNA Synthetase. Mutations in the double allele of cytoplasmic isoleucine-tRNA synthetase (IARS) have been shown to cause prenatal onset syndrome (GRIDHH) with symptoms such as growth retardation, impaired mental development, hypotonia and liver disease [45]. One of the core manifestations of PD is increased muscle tone, suggesting that *iars* may play a protective role in PD. GSEA analysis showed that *iars* were mainly enriched in lysosomal and immune-related pathways. *ttc*19 encodes a protein with a tetrapeptide repeat (TPR) domain. This protein is involved in the formation of mitochondrial respiratory chain III. It may also play a role in cytoplasmic division [46].

Abnormal mitochondrial function is associated with Parkinson’s disease [47]. This provides a theoretical basis for *ttc*19 as a Pd hub gene. GSEA analysis also showed that *ttc*19, and *dld* were enriched in mitochondria-related signaling pathways such as the TCA cycle. GSVA analysis showed that *dlk*1, *iars* and *ttc*19 were all associated with glycosphingolipid biosynthesis at low expression. Sphingolipids regulate key aspects of brain function are potent modulators of inflammatory processes, and were found to play an important protective role in Parkinson’s disease by Belarbi Karim et al [48]. All of these findings point to the importance of glycosphingolipid biosynthesis in PD and point the way to the next step in research.

We built a PD classification diagnostic model using a logistic regression algorithm using four hub genes, *dld*, *dlk*1, *iars* and *ttc*19. We validated the accuracy of these four hub genes and diagnostic models in four publicly available independent datasets. The mSVM-REF and LASSO algorithms have been widely used for data analysis. We used an extension of this algorithm, mSVM-RFE, to further improve the model prediction accuracy [49]. Two types of machine learning also have many applications in clinical settings [24]. There is precedent for WGCNA combined with machine learning algorithms to screen for disease signature genes [50]. However, it is worth noting that no studies have been conducted in the PD domain using this combination of WGCNA combined with mSVM-RFE and LASSO machine learning.

It is also worth noting the robustness of our method. We can notice from the ROC curves that the single-gene model may have an overfitting problem in the original dataset, but through the use of the combined diagnostic model, we successfully customer service this problem and achieve a performance on the validation set that is comparable to the original training set. This confirms that the combined diagnostic models obtained using WGCNA in conjunction with machine learning have high reliability and generalization capabilities despite differences such as experimental locations, experimenters, and experimental platforms between different datasets. Therefore, WGCNA combining two machine learning algorithms to identify PD immune-associated hub genes is a bold attempt to provide an algorithmic-level perspective for understanding the mechanisms at the genetic level of PD. Our study shows that four hub genes are involved in regulating immune, lysosomal, metabolism-related pathways, energy metabolism pathways, vesicular transport and synaptic pathways. This also suggests that Parkinson’s disease is a heterogeneous disease resulting from a complex interaction of multiple factors. Also, immunoassays confirmed that the hub gene is closely associated with immune cell infiltration, ensuring that dysregulation of the immune microenvironment plays a crucial role in the pathogenesis of PD.

Recent studies have shown that alterations in ncRNA(non-coding RNA,ncRNA) are associated with the development of PD [51–53]. Multiple ncRNAs have been experimentally demonstrated to be involved in pathological processes such as apoptosis, α-syn misfolding and aggregation, mitochondrial dysfunction, and autophagy in Parkinson’s disease [54]. Many studies based on brain tissues and body fluids from PD patients suggest that variants in ncRNAs and their target genes may trigger or exacerbate neurodegenerative pathogenesis and serve as potential non-invasive biomarkers for PD [55]. To this end, we constructed a ceRNA regulatory network based on four hub genes, with the hope that practical non-invasive biomarkers or intervention targets could be identified in future studies. Among them, SNHG14 (small nucleolar RNA host gene 14, SNHG14) has been shown to attenuate dopaminergic neuronal damage in PD through targeted knockdown [56]. Possible mechanisms are through the reduction of apoptosis and inflammatory responses, among others, which point to a promising target for PD intervention and treatment. In addition, hsa-miR-590-3p was significantly downregulated in the MPP+treated cell model. The findings suggest a possible involvement in the regulation of mitochondrial function and ROS(Reactive Oxygen Species, ROS) levels and thus in the development of PD [57]. hsa-miR-129-5p has also been found to be differentially expressed in both AD and PD and is possibly involved in the development of Huntington’s disease, suggesting that this gene may be involved in a common pathogenic mechanism of neurodegenerative diseases, although the exact mechanism is not yet clear [58]. We also expect that future related studies can refer to the ceRNA network we constructed.

Of course, there are many shortcomings in our work. First, the lack of a large sample to support the Parkinson’s disease gene chip database compared to Alzheimer’s disease also affects the accuracy of our data. In addition, the database provided incomplete information about the samples, such as the stage of disease of the samples, which limited the possibility of more precise analysis based on the staging, and at the same time produced some errors in our results. We hope to obtain more complete sample information in future studies. Second, our study samples were all autopsy samples from deceased patients, and the model has limitations in terms of clinical translation. Although algorithm-level validation was performed in this study, biological-level validation was not performed, which also reduces the credibility of the results. Third, machine learning algorithms such as LASSO and mSVM-RFE were used in this study, but other algorithms such as random forest were not used in this study. We hope to use multiple machine learning algorithms to improve the research methodology in future studies. Fourth, some PD patients have a combination of other neurodegenerative lesions, which affects the model’s accuracy. We hope to improve on this in the following study.

Despite these limitations, we believe our study is still scientifically valuable for understanding gene expression changes in PD patients. These results could provide insight into the molecular mechanisms of disease. In addition, they can provide clues about potential therapeutic targets for future research.

## 5. Conclusion

We used WGCNA combined with two machine learning algorithms for feature screening and combined with immune cell infiltration analysis to identify potential PD immune-related hub genes. Meanwhile, a ceRNA regulatory network was constructed based on four immune-related hub genes, and a logistic regression classification diagnostic model for PD was established. We validated the accuracy of the hub genes and the accuracy of the model using three independent datasets. The results show that the model can distinguish PD samples from healthy samples. In this study, new immune-related hub genes were identified and a new model for classification and diagnosis was developed. PD hub genes that may be involved in the regulation of lysosomal, immune and synaptic functions are introduced.

## 6. Research contribution

### 6.1 Identify hub genes

We successfully identified four hub genes associated with Parkinson’s disease (PD), namely *dld*, *dlk*1, *iars*, and *ttc*19, through a combination of differential expression analysis, WGCNA, and machine learning algorithms (LASSO and mSVM-RFE). This finding provides new insights into the pathogenesis of PD. Provide a theoretical basis for exploring possible drug targets.

### 6.2 Diversity of Parkinson’s disease mechanisms revealed

Our research is not limited to a single biological process, but also reveals the potential role of these hub genes in multiple domains. This includes energy metabolism, the immune system, cellular lysosomal function, neuronal transmission, and many other aspects. This highlights the fact that PD is a complex multifactorial disease involving multiple biological processes.

### 6.3 Modeling classification

We successfully constructed a PD categorical diagnostic model based on four hub genes (*dld*, *dlk*1, *iars*, and *ttc*19). This offers a potential method for early diagnosis of PD and provides a basis for individualized treatment.

### 6.4 Exploring future research directions

Our study offers several potential directions for future PD research, including biological validation, more comprehensive sample data, application of other machine learning algorithms, and clinical translational research. This will help deepen our understanding of PD and find more effective treatment strategies.

Through these contributions, our study provides new perspectives and tools to the field of PD research, which is expected to have a positive impact on the early diagnosis and treatment of PD.

## Supporting information

S1 Appendix The specific results of differential expression analysis are shown in S1 Appendix. (XLS)

S2 Appendix GS and MM calculation results for each gene. (XLS)

S3 Appendix The specific GO and KEGG enrichment results of DEGs are integrated into S3 Appendix. (XLS)

S4 Appendix The specific GSEA and GSVA enrichment results for each gene are integrated into S4 Appendix. (XLS)

S5 Appendix The immune infiltration fraction of 28 immune cells in the samples. (XLSX)

S6 Appendix The specific details of the ceRNA network are shown in S6 Appendix. (XLSX)
